# Exploring Acute Pancreatitis Clinical Pathways Using a Novel Process Mining Method

**DOI:** 10.3390/healthcare11182529

**Published:** 2023-09-13

**Authors:** Xue Yang, Wei Huang, Weiling Zhao, Xiaobo Zhou, Na Shi, Qing Xia

**Affiliations:** 1 Department of Pancreatic Surgery and West China Biomedical Big Data Center, West China Hospital, Sichuan University, Chengdu 610041, China; xxer99@163.com; 2 Pancreatitis Center, Department of Integrated Traditional Chinese and Western Medicine, West China Hospital, Sichuan University, Chengdu 610041, China; wei.huang.scu@vip.163.com; 3 Center for Computational Systems Medicine, School of Biomedical Informatics, The University of Texas Health Science Center at Houston, Houston, TX 77030, USA; weiling.zhao@uth.tmc.edu (W.Z.); xiaobo.zhou@uth.tmc.edu (X.Z.)

**Keywords:** healthcare, process mining, clinical pathway, acute pancreatitis, electronic medical record

## Abstract

Mining process models of medical behavior from electronic medical records is an effective way to optimize clinical pathways. However, clinical medical behavior is an extremely complex field with high nonlinearity and variability, and thus we need to adopt a more effective method. In this study, we developed a fuzzy process mining method for complex clinical pathways. Firstly, we designed a multi-level expert classification system with fuzzy values to preserve finer details. Secondly, we categorized medical events into long-term and temporary events for more specific data processing. Subsequently, we utilized electronic medical record (EMR) data of acute pancreatitis spanning 9 years, collected from a large general hospital in China, to evaluate the effectiveness of our method. The results demonstrated that our modeling process was simple and understandable, allowing for a more comprehensive representation of medical intricacies. Moreover, our method exhibited high patient coverage (>0.94) and discrimination (>0.838). These findings were corroborated by clinicians, affirming the accuracy and effectiveness of our approach.

## 1. Introduction

In the past few decades, the intensification of population aging and the continuous rise in healthcare costs have imposed pressures on healthcare systems and resources. Traditional healthcare models often suffer from issues such as wastage of medical resources and low efficiency, making it challenging to meet the modern society’s demand for high-quality and efficient medical services. To enhance healthcare efficiency, optimize resource allocation, and ensure healthcare quality, it is necessary to reevaluate and redesign the healthcare processes. This involves delineating clear diagnostic and treatment pathways, thereby breaking down intricate medical procedures into organized and standardized steps. As a result, the concept of clinical pathway (CPA) [1] has emerged, which refers to a set of standardized treatment patterns and protocols established for a specific disease, aiming to optimize healthcare services guided by evidence-based medical evidence and guidelines. Countries like Sweden, the Netherlands, and the United Kingdom have integrated clinical pathways into their standardized medical practices. These practices have shown that clinical pathways aid in regulating and guiding the treatment processes of a variety of medical conditions, thereby reducing unnecessary medical costs and time, while enhancing medical quality standards. With the application of clinical pathway management, many problems are inevitably exposed. First, most modern medical processes require multidisciplinary cooperation, while the existing clinical pathway standards are process standards for single disease treatment and care services, which are fixed and static. Secondly, the differences in population and environment in different regions will also cause variations in the implementation of clinical pathways, which brings great difficulties to the implementation of clinical pathways. In addition, most clinical pathways are based on evidence-based medicine and developed by experts through discussion. The subjective influence of experts can be great [2]. These problems have caused serious obstacles to the further development of clinical pathways, and the clinical pathway diagnosis and treatment plan needs to be continuously improved and optimized.

With the progress of medical treatment and the development of big data technology, many medical records in the HIS system are analyzed, providing an interesting direction for the exploration and optimization of clinical pathways [3]. In recent years, the increased application of process mining in clinical practice has been extended to emergency care [4,5,6], tumor care [7,8,9], telemedicine [10], COVID-19 [11,12], and other disciplines, confirming the great potential of process mining technology in clinical pathway analysis and optimization.

However, the clinical and nursing domains are extremely complex compared with other industry processes. Firstly, medical behavior is nonlinear, repeatable, and unpredictable. Secondly, clinical and nursing decisions need to consider the individual differences in patients, while the traditional process model has poor variability. In addition, detail differences should be considered in clinical decision-making. For example, for many acute diseases, the first 2~3 weeks of nursing are very important. The nursing behavior at different stages may be similar, but the nursing grade and the frequency of medical operation might be different, which is more noteworthy information for medical personnel. Unfortunately, existing relevant works have not analyzed these details. Therefore, it is necessary to design a new process mining algorithm, optimizing the expression of the process model to better support medical decision-making.

To solve the problems of process mining in the clinical and nursing fields mentioned above, we propose a novel process mining method for complex clinical pathways. Our method aims to preserve important details in medical procedures, not only expressing the clinical pathways of the most common patients, but also expressing the treatment patterns of uncommon patients, rather than discarding them from the process model. Furthermore, the process model should be concise and interpretable to clinicians.

## 2. Related Works

### 2.1. Process Mining Techniques in Healthcare

To provide data-based support for specific medical processes, many scholars have tried different process mining technologies such as alpha algorithm [13], fuzzy miner [14], heuristic miner [15], and inductive miner [16]. Among them, inductive miner is the most widely used one. These process mining technologies are used together with ProM or other process mining tools to generate a visualized medical event sequence in a graphical way. In the past few decades, more and more scholars have devoted themselves to the research of process mining in the field of healthcare and have achieved a series of results [10,17]. However, because the medical and nursing events recorded in the HIS system are often complex and changeable, the clinic and nursing service are extremely complex domains with many characteristics that business and industry processes do not have [18]. Log-based mining usually leads to the inevitable spaghetti effect [19] in most medical process models. Such medical process models are extremely difficult to understand and cannot provide usable knowledge for medical professionals.

To simplify the medical process model and avoid the high variability of the clinical pathway, some scholars have proposed local model extraction schemes [8,20,21,22]. However, the local model ignores the influence of medical events in the whole process. In the clinical pathway, the time range of medical operations is also an important factor in the rehabilitation of patients. Some studies focused on a group with similar behaviors, such as selecting only a small group of patients with similar medical problems [23,24]. Although this method could reduce the complexity of the models, different groups of medical events cannot be combined, and the complexity would not be reduced.

### 2.2. Clustering Techniques

In medical process mining, clustering technology is generally used in data preprocessing. The quality of clustering directly affects the results of process mining, and the key lies in the interpretability of the clustering results. Some scholars [25] tried to realize the unsupervised clustering of medical events by constructing different features. However, the clustering results vary greatly with the parameters. How to find the most reasonable parameters has not been well resolved. At the same time, it is difficult to explain the clusters, so the clustering operations will reduce the comprehensibility and variability of the process model. Some researchers [26,27] used the classification system of the medical field itself (such as ICD9) to classify medical events. Although this classification method is professional and easy to understand, it is based on the management classification. However, the medical process model of the clinical pathways ought to be based on the treatment purpose classification. Vathy-Fogarassy et al. [7] integrated expert knowledge and performed medical sequence analysis in a semi-automatic manner. The results showed that the classification based on expert knowledge was not a time-consuming and expensive task, and it could be easier for retaining key information.

### 2.3. Existing Problems and Our Improvements

Despite numerous attempts at utilizing process mining and clustering technologies, the research on medical process mining is far from perfect. There are several challenges associated with creating process models for such events:

1. The medical events are usually nonlinear, repeatable, and unpredictable, making the process models prone to the “spaghetti effect”.
2. In clinical decision-making, it is essential to consider the differences in treatment details. However, the existing relevant works have failed to analyze these particulars. The primary factor contributing to these differences lies in the level or frequency of most medical events.
3. Common medical process models typically mine common paths aligned with the most frequent patients, and medical events in these models are also the most common ones. However, standard medical processes are not always interesting, and medical professionals are sometimes more willing to discover uncommon medical processes.

To tackle these challenges, we have developed a method called the “Fuzzy Process Mining Method” (FPM Method). Our approach aims to comprehensively explore and simulate significant details (including uncommon events) of medical events in clinical pathways, while emphasizing the differences in the level or frequency of long-term sustained medical events. Our method incorporates the following specific improvement measures:

1. In order to eliminate the spaghetti effect, we distinguish between continuous long-term medical events and discrete temporary medical events based on the characteristics of medical events, and then establish a multi-level expert classification system.
2. To delve deeper into the details of medical events, we use fuzzy values to express the level or occurrence probability of medical events, which enables more precise expressions of uncommon medical events.
3. For temporary medical events, we implement several measures, mainly including setting a “Flexible Time Window”, merging similar medical events based on “Weighted Jaccard Similarity”, hierarchical clustering based on “Euclidean Distance”, and so on. In this way, the nodes of the process model can express the details of medical events in a hierarchical manner. For continuous long-term medical events, we use statistical methods to analyze the dynamic changes in the degree or frequency of medical event execution.
4. Extensive experiments conducted on various types of acute pancreatitis datasets from a large general hospital demonstrate the effectiveness, high interpretability, and superiority of the proposed method.

## 3. Methodology

**Figure 1:**
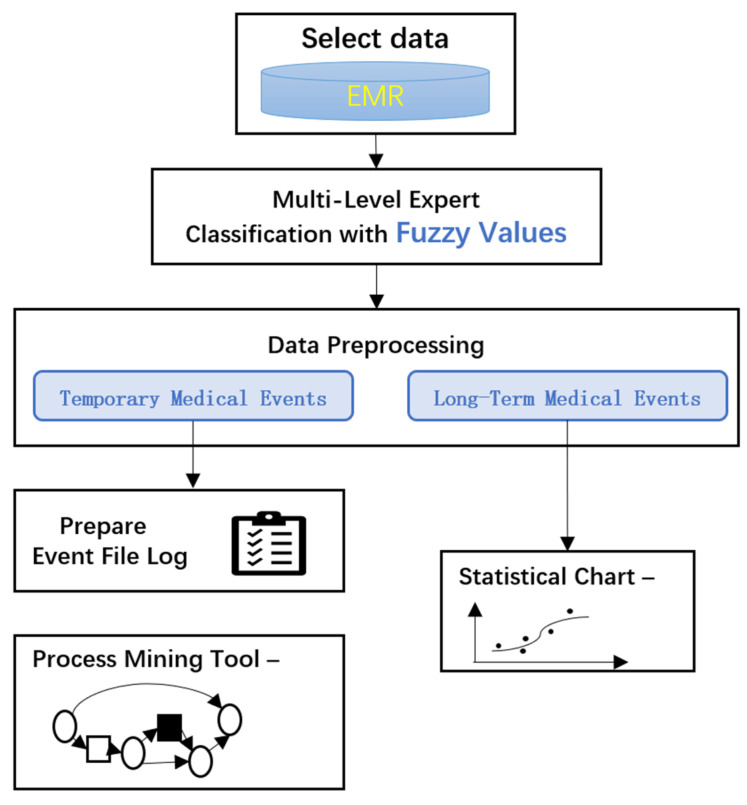
Flowchart of the proposed scheme.

The overall framework of the fuzzy process mining method is shown in Figure 1. First, the raw EMR data are screened, filtered, and cleaned. Second, a multi-level expert classification system with fuzzy values is used to transform the data, and medical events are divided into long-term events and temporary events according to the effectiveness of the clinical pathway. Third, the long-term and temporary medical events are processed separately. Finally, the process model is generated by the process mining tools, and the statistical results are output.

### 3.1. Select Data

In the EMR medical event log, the clinical diagnoses, treatments, and nursing information of patients are recorded, including the basic information of patients, medical examination records, doctor’s orders, nursing records, and so on. In the clinical pathways, these types of information are very important for the treatment and rehabilitation of patients, especially the doctor’s orders and nursing records. Moreover, since the patients usually have more than one disease, we should only select the patients whose “main diagnosis” is the disease to be studied.

To further simplify the data, we only select the key types of medical events for analysis. In general, at least the following information needs to be covered.

1. Basic patient information, including the patient’s ID, age, gender, etc.
2. Diagnostic information, including the main diagnosis and complications.
3. Medical event information, mainly including key medical orders and nursing operations.
4. Time stamp information of medical records (generally accurate to “day”).

Exclusion criteria:

1. Patients whose treatments are significantly different from the general patient group, such as minors or pregnant patients.
2. Those with other serious disease cases.
3. Irrelevant or unimportant records.
4. Cases with abnormal admission or withdrawal, such as midway transfer or for personal reasons.
5. Errors (such as null values) and duplicate records.
6. Nodes with too few total occurrence times to eliminate noisy data.

### 3.2. Multi-Level Expert Classification with Fuzzy Values

The raw medical events are sometimes too detailed, which makes it difficult to generalize a common conclusion about the treatment mode, and the process model becomes complicated and difficult to understand. This is the so-called “spaghetti effect”. The process model can be reasonably simplified by classifying medical events in multi-levels. Expert classification ensures the accuracy and expertise of the classification results, and multi-level is a prerequisite for obtaining medical events with different levels of granularity. Additionally, given the variations in treatment approaches for different patients, using fuzzy values to express the frequency or probability of medical events can effectively preserve the details of medical procedures, thereby ensuring precision and interpretability in the final outcomes.

**Figure 2:**
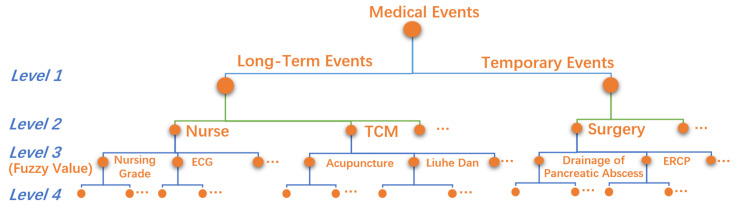
Multi-level expert classification with fuzzy values.

The multi-level expert classification system has four layers, as shown in Figure 2.

Level 1. It is divided into two groups: temporary medical events and long-term medical events.

Level 2. High-level medical classes, such as surgery, nurse, etc.

Level 3. Low-level medical classes, such as ERCP, ECG, etc.

Level 4. The raw medical event records, such as blood pressure measurement, heart rate measurement, etc., are not suitable for direct analysis.

It can be seen from the multi-level classification system that the data used for process mining are accurate to low-level medical classes in Level 3, which are the most critical medical classes for clinical pathway mining. To deeply explore the essential details of disease treatment and care, we set a fuzzy value attribute for each Level 3 medical class to represent the degree, probability, or frequency information of medical operations. For example, the “nursing grades” contains “super care”, “primary care”, “secondary care”, and “tertiary care”, so the “value” is set as 1, 0.75, 0.5, or 0.25, respectively, to distinguish the degree of nursing. For medical events without details, the “value” is set as 0 or 1.

Let the medical event $E=\left(e,t,p,y\right)$, in which e,t,p,y represents the class of the medical event in Level 3, the date, the fuzzy value, and the group in Level 1 (long-term event or temporary event); then, the medical event record of patient $P_{i}$ is defined as

(1) $$P_{k}=\left[\begin{array}{l} \left(e_{1},t_{k1},p_{k1},y_{1}\right),\\ \left(e_{2},t_{k2},p_{k2},y_{2}\right),\ldots ,\left(e_{N},t_{kN},p_{kN},y_{N}\right) \end{array}\right],$$

where *k* represents the index of patient and *N* represents the number of medical classes in Level 3.

### 3.3. Data Processing

For long-term medical events, we primarily focus on their variations in intensity; for temporary medical events, our main emphasis is on their occurrence probabilities and sequence. Therefore, after mapping the EMR data to the multi-level expert classification system with fuzzy values, different data processing flows are performed for long-term events and temporary events.

#### 3.3.1. Data Processing of Temporary Medical Events

Considering that the Level 2 event classes are relatively independent, we carry out the data processing flow of temporary medical events for each Level 2 medical class separately.

It can be considered that the combination of medical events received by patients every day is usually similar over a period. These combined medical events are important for clinical treatment and nursing, which cannot be separated for processing. In addition, many medical events often include time-indeterminacy, leaping, and repeatability, increasing the complexity of clinical pathways. To solve this problem, we define an “Elastic Time Window” to package a series of Level 3 medical events that belong to the same category as the Level 2 class by calculating the similarity between two adjacent dates. When we merge medical events within an Elastic Time Window, we consider that the purpose of the medical events within the Elastic Time Window is relatively stable and similar.

**Figure 3:**
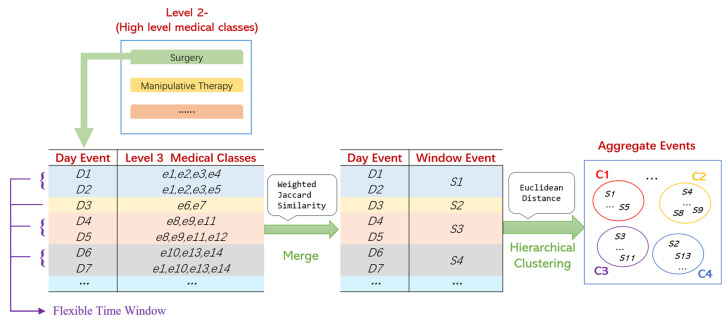
Data processing of temporary medical data.

The detailed data processing steps are shown in Figure 3.

The data processing of temporary medical events includes 6 steps.

Step 1. All Level 3 medical events of a patient in one day are regarded as a “Day Event”, and the “Jaccard Weighted Similarity” between the adjacent Day Events is calculated. By assigning higher weights to the important medical events, the impact of medical events on the disease can be considered more reasonably. The “Jaccard Weighted Similarity” $Simi_{i,i+1}^{}$ can be defined as follows:

(2) $$Simi_{i,i+1}^{}=\frac{\left|D_{i}\cap D_{i+1}\right|}{\left|D_{i}\cup D_{i+1}\right|},$$

where *i* is the index of the hospitalization day and $D_{i}$ is the Day Event of Hospitalization Day *i*.

Step 2. Let the threshold be λ. If $Simi_{i,i+1}^{}\geq \lambda$, these adjacent Day Events are grouped together, and we call this group a “Flexible Time Window”. It can be considered that medical decisions remain stable within a Flexible Time Window.

Step 3. Day Events are merged into a Flexible Time Window by the “average” method, then the merged medical event is called a “Window Event”, which is defined as follows:

(3) $$S=\frac{\sum_{T_{1}\leq t<T_{2}}D_{t}}{T_{2}-T_{1}+1},$$

where $T_{2},\;T_{1}$ is the Begin Time and End Time of the Window Event, respectively, and $T_{2}>T_{1}$. $D_{t}$ is the vector of the Day Event.

Similar Day Events are merged into a Flexible Time Window, and 0–1 fuzzy values are used as the weights of medical classes, representing the probability of their occurrence. Therefore, there will be no information loss. For example, in Figure 3, if *D*_4_ {*e*_8_, *e*_9_, *e*_11_} and *D*_5_ {*e*_8_, *e*_9_, *e*_11_, *e*_12_} are merged into *S*_3_ {*e*_8_(1.0), *e*_9_(1.0), *e*_11_(1.0), *e*_12_(0.5)}, the fuzzy values reflect the frequency of medical events.

Step 4. All the Window Events are aggregated by the hierarchical clustering method (metric: “Euclidean”, method: “Average”), and then we can obtain *M* clusters $\left\{C_{1},C_{2},C_{3},\ldots ,C_{M}\right\}$, which are called “Aggregate Events”. We can presume that an Aggregate Event represents a series of Window Events with similar medical purposes and effects.

Step 5. We average the fuzzy value of the Level 3 medical events in each Aggregate Event to gain the probabilities of the Level 3 medical events, providing accurate information for the medical workers.

Step 6. We consider those Aggregate Events generated by very few unusual clinical pathways as noise and remove them to further simplify the results.

#### 3.3.2. Data Processing of Long-Term Medical Events

The long-term medical events should follow the following steps:

Step 1. Convert the time stamp information of the date format to the indexes of hospitalization days.

Step 2. Normalize the fuzzy values of the Level 3 medical events.

Step 3. Average the fuzzy values by the hospitalization days to indicate the probability of Level 3 temporary medical events.

After the data processing steps above, we can obtain relevant statistical charts to analyze the changes in different Level 3 long-term medical events with the hospitalization day.

### 3.4. Prepare Event File Log and Generate Process Model

Process mining is only suitable for temporary medical events. Before process mining, it is necessary to sort out the results obtained from the previous data processing and formulate an event log for process mining. In the event log, at least the following information must be included.

1. Patient ID: A unique identifier for the patient.
2. Event Name: The name of the aggregation event. The information contained in each type of Aggregate Event includes a series of Level 3 medical events and the corresponding probabilities.
3. Start and End Time: Since the Aggregate Event is a collection of similar Window Events, the Start Time and the End Time of an Aggregate Event are the start date and end date of the corresponding Window Events $T_{1},\;T_{2}$.

After preparing the event log, we import it into the ProM 6.12 process mining software, and use the inductive miner process mining technology to obtain the petri net process model.

## 4. Application of Acute Pancreatitis and Data Description

Acute pancreatitis is a common acute abdominal issue in general surgery. This disease is characterized by a rapid onset, dangerous conditions, complexity, and multiple complications. Significant individual differences have been shown during treatment, especially in severe acute pancreatitis. On the one hand, the existing evidence-based clinical pathway guidelines for acute pancreatitis often cause variations in clinical pathways due to the different complications of patients, resulting in uncertainty and randomness in medical operations. On the other hand, the existing process mining methods often appear to have a spaghetti effect, which is challenging to apply to the clinical pathway of acute pancreatitis. Therefore, in this study, we applied the proposed method to the clinical pathway mining of acute pancreatitis to support the clinical treatment of acute pancreatitis. Our results demonstrated the superiority of the proposed method.

We collected the EMR data of patients who received a diagnosis and treatment for acute pancreatitis during outpatient or inpatient treatment from 2009 to 2018 from a large general hospital. The treatment of acute pancreatitis in this hospital is at the international leading level, and it is recognized as a model in treating critical and severe cases. The results obtained from this dataset will provide important references and guidance for the clinical treatment of acute pancreatitis in other medical institutions.

The total number of patients included in this dataset is 22,602, and the basic information, examinations, consultations, medical orders, nursing information, and other related information of patients are recorded. Recording time is usually accurate to the day. Among those data, medical orders and nursing information are comprehensive and fine-grained. During the data cleaning phase, we excluded events not directly related to the treatment of acute pancreatitis. In addition, we only selected patients with a primary diagnosis of acute pancreatitis, and excluded cases of patients under 18, pregnant patients, and cancer patients. Due to the large differences in the treatment of different types of patients with acute pancreatitis, we further divided the symptoms of acute pancreatitis into three sub-datasets, including 10,411 mild cases, 3790 general severe cases, and 1888 severe cases accompanied by multiple organ failure (MOF).

According to the preliminary analysis of the treatment process of acute pancreatitis, most patients will get better within 40 days after admission, while many critically ill patients will be transferred, die, or withdraw themselves after 40 hospitalization days. Therefore, we set the maximum observation period to 40 days.

To preserve the treatment details of acute pancreatitis while improving the simplicity of the process model to avoid the spaghetti effect, we generated a process model for each Level 2 medical class of temporary medical events.

## 5. Results and Discussion

### 5.1. Main Results of Severe Acute Pancreatitis

**Figure 4:**
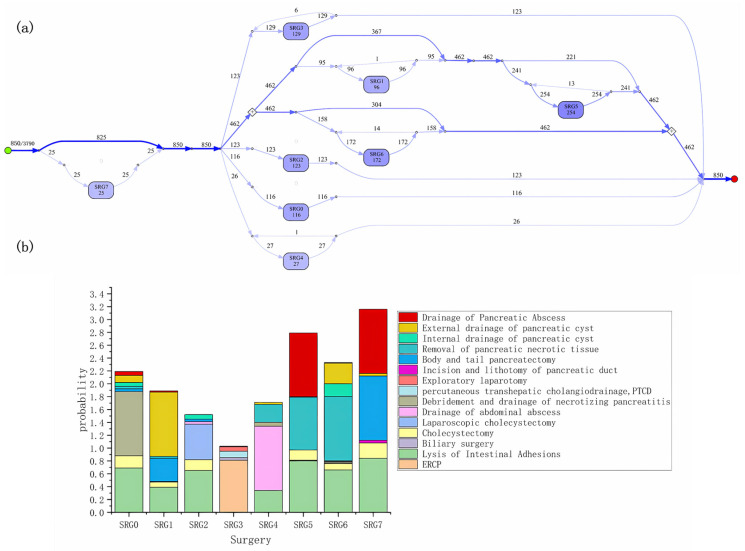
(**a**) The surgery process models of the severe acute pancreatitis; (**b**) the combinations of surgeries with different frequencies in each type of node.

By mining and analyzing the severe acute pancreatitis dataset, we obtained easy-to-understand results, highlighting the key treatment and nursing characteristics of the disease, as is shown in Figure 4a. Furthermore, to eliminate the noise caused by very few cases in the dataset, we excluded nodes with occurrence times less than 10 in the process model.

From the process model, among 3790 patients with severe acute pancreatitis, 850 patients received surgical treatment. The numbers in line segments and nodes represent the number of patients passing through the path or node. The darker the color of the lines and nodes, the higher the probability of occurrence of the corresponding medical event combination. A few patients received surgical treatment again in a period. In addition, each type of node includes several different types of surgeries. Figure 4b shows the combinations of surgeries with different frequencies in each type of node. High-probability surgery reflects the commonality of severe acute pancreatitis treatment, while low-probability surgery is the best reflection of individual differences in patients. Among 850 patients with severe acute pancreatitis who underwent surgery, the most common surgical combination is SRG5 (254 cases), mainly including the drainage of pancreatic abscess, removal of pancreatic necrotic tissue, lysis of intestinal adhesions, and cholecystectomy.

**Figure 5:**
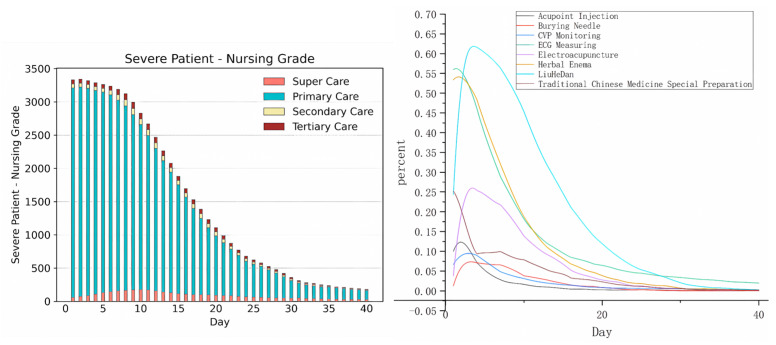
The changes in long-term medical events over time after admission.

Daily nursing care and TCM treatment belong to long-term medical events. For such events, we mainly study these changes over time after admission. Figure 5 shows the monitoring, TCM treatment, and the probability changes of different nursing grades for patients with severe acute pancreatitis within 40 hospitalization days. It is not difficult to observe that most patients with severe acute pancreatitis experience a hospital stay of 30 days, with a particular focus on the initial two weeks after admission, which constitutes a critical phase in their treatment. For most patients with severe acute pancreatitis, the key observation period falls between two weeks and thirty days after admission. A minority of patients may require re-operation during this observation period.

Compared with the traditional process model, our method has the following important characteristics:

Firstly, traditional process models can only depict the most common event patterns using the color depth of nodes, resulting in overly generalized summaries of medical events. In contrast, the nodes in our process model encompass combinations of more intricate medical events, enabling the reflection of detailed information and enhancing their value.

Secondly, the fuzzy values in our process model reflect the degrees or probabilities of medical operations, which aligns more closely with the actual scenarios of medical procedures. Obviously, the traditional process models cannot achieve such an effect.

Thirdly, different analysis processes ought to be conducted to distinguish long-term sustained medical events from temporary ones. Such processes align with clinical practice and help to express key details reflected in the clinical pathway. These details include the categories and probabilities of various medical events, as well as the frequency changes of long-term medical events with hospitalization days. The possible spaghetti effect in our process models is properly avoided by distinguishing statistical charts representing long-term medical events from the process model representing temporary medical events, which the traditional process models cannot achieve.

### 5.2. Comparative Analysis and Discussion

**Figure 6:**
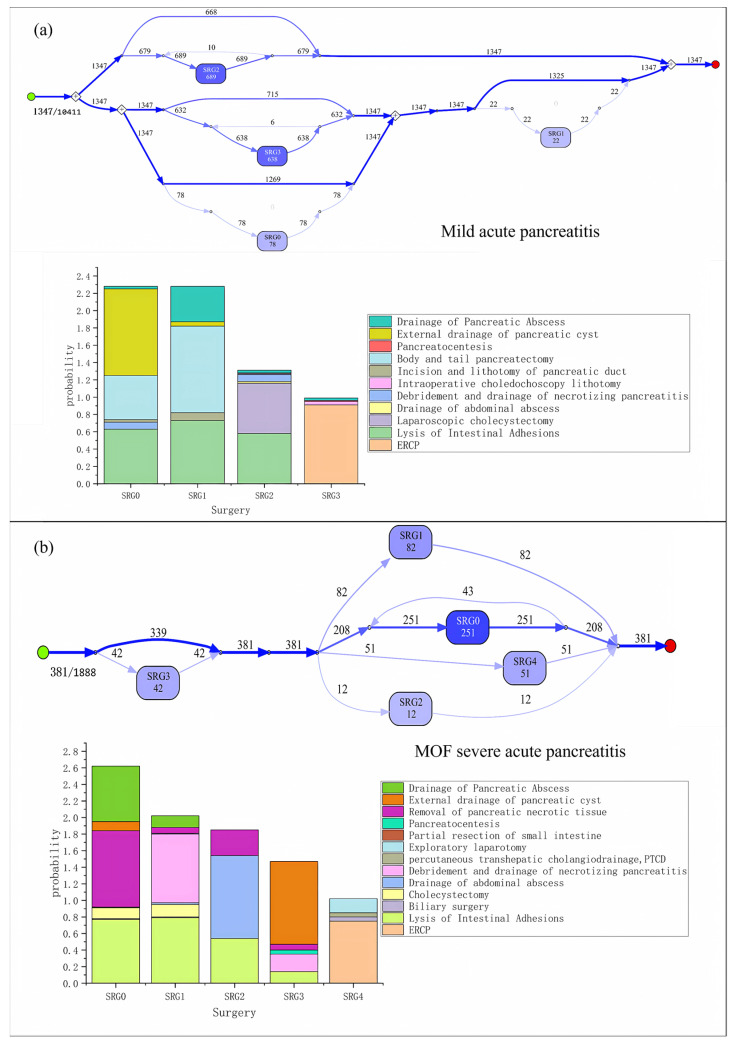
(**a**) The surgery process models of mild acute pancreatitis; (**b**) the surgery process models of MOF severe acute pancreatitis.

In this section, we will further obtain the results of different types of acute pancreatitis and compare them. The process models of surgical procedures for mild and MOF severe acute pancreatitis are shown in Figure 6.

**Figure 7:**
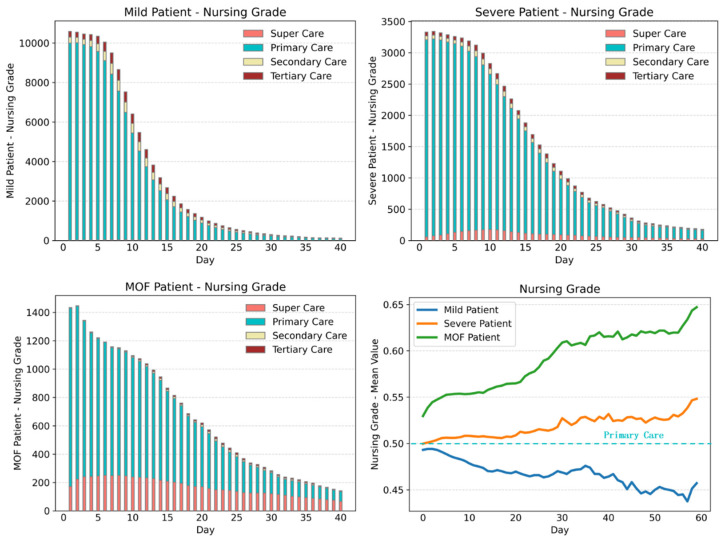
Nursing grade change charts for three types of acute pancreatitis.

**Figure 8:**
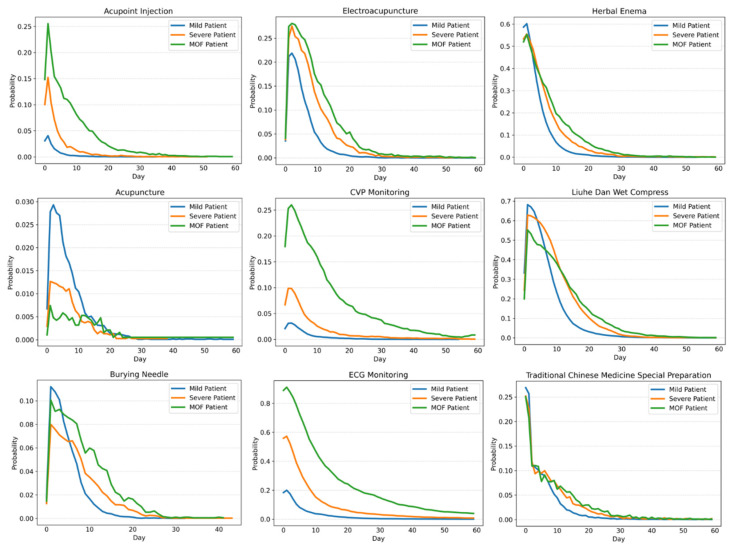
Change charts of monitoring items and TCM treatment operations for three types of acute pancreatitis.

The comparisons of nursing grade change charts for three types of acute pancreatitis are shown in Figure 7 and Figure 8.

Comparing the results of three groups of acute pancreatitis patients with different symptoms, we can obtain the following information:

1. The surgery proportion of severe acute pancreatitis patients is significantly higher than that of mild acute pancreatitis patients.
2. The hospitalization days of most mild patients, severe patients, and MOF severe patients are 20, 30, and 40 days, respectively, and the nursing grade is mainly primary care. With the aggravation of the disease, the proportion of super care is also increased accordingly. However, with the increase in hospitalization days, the nursing grades of the two types of severe patients show an upward trend, especially for that of MOF severe patients. Mild acute pancreatitis patients can easily recover, while the severe acute pancreatitis patients are likely to get worse or even die within a period.
3. From the comparison of nursing grade, monitoring items, and TCM treatment operations, we can conclude that the first 10 hospitalization days are particularly critical for the treatment of acute pancreatitis, which reflects the obvious characteristics of integrated traditional Chinese and Western medicine treatment. After 10 hospitalization days, most mild patients will markedly improve, followed by a period of rehabilitation and stability. However, for severe patients and MOF severe patients, extending the critical treatment period is also in line with the actual clinical treatment situation.
4. For patients with different types of symptoms, the similarities, and differences in the combination of surgical types can also be seen in the process model of surgical operation. Table 1 shows the specific proportion of the most common surgical operations among the three groups of patients.

It can be seen from Table 1 that mild acute pancreatitis is characterized by mild symptoms, and most of the surgeries are minimally invasive operations such as ERCP and laparoscopy, while the most common surgeries for severe patients are similar to those of MOF patients. Surgeries mainly include “removal of pancreatic necrotic tissue” and “lysis of intestinal adhesions”, and catheter drainage is also needed. For most MOF severe patients, the “drainage of pancreatic abscess” is the main method of catheter drainage.

Based on the experimental results, we can draw the following conclusions:

1. Through comparing treatment methods for different types of acute pancreatitis, we further demonstrated that our proposed method could provide clear explanations and analyses of medical events in clinical pathways. Additionally, we have discovered differences among different types of acute pancreatitis clinical pathways. This indicates that our method has strong interpretability.
2. The experiment generated aggregated medical events, such as SRG2 and SRG3, which represent some of the most common medical events. These aggregated medical events cover a portion of small-probability medical events, such as “drainage of pancreatic absence” and “incision and liturgical of pancreatic duct”. That is, our method simulated small probability medical events in a hierarchical manner in the process models. This improvement demonstrates strong fine-grained information mining capabilities compared to previous works.

### 5.3. Sensitivity Analysis

In our proposed method, we need to cluster Window Events by a hierarchical clustering method to obtain Aggregate Events. As we all know, the clustering parameter *t* is a critical factor for the clustering effect, and the clustering effect is also a key factor for the quality of the process model. In this section, we will perform a sensitivity analysis of the clustering parameter *t*, and then verify the reliability and effectiveness of the method we have proposed.

Based on the fuzzy process mining method, we believe that a good process model should have two characteristics. First, if we define a few cases as noise, the proportion of noise should be as small as possible, so the process model has high patient coverage and discrimination. Secondly, the difference between clusters should be obvious.

To evaluate the clustering results, the discrimination is defined as follows:

**Definition** **1.** *Let the Aggregate Event* $C_{i}=\left\{\left(s_{1},p_{1}\right),\left(s_{2},p_{2}\right),\ldots ,\left(s_{N},p_{N}\right)\right\}$*, where* $\left(s_{1},s_{2},\;\ldots ,\;s_{N}\right)$ *is the feature vector,* $\left(p_{1},p_{2},\;\ldots ,p_{N}\right)$ *is the fuzzy value vector, and N is the number of features, then the discrimination between two Aggregate Events* $C_{i}$*,* $C_{j}$* is defined as follows:*

(4) $$\begin{array}{l} Dis\left(i,j\right)=\max\\ \left(\left|p_{i1}-p_{j1}\right|,\left|p_{i2}-p_{j2}\right|,\ldots ,\left|p_{iM_{k}}-p_{jM_{k}}\right|\right), \end{array}$$

*and the discrimination of the final process model is defined as follows:*

(5) $$Dis=\frac{2\sum_{0\leq i<j<N}Dis\left(i,j\right)}{\left(N-1\right)N}$$

*where N is the number of Aggregate Events.*

If we change the hierarchical clustering parameter t, we can obtain the values of coverage and discrimination, which are shown in Table 2.

Obviously, the parameter *t* is inversely proportional to the number of clusters and discrimination size, and is directly proportional to patient coverage. If parameter *t* = 0, it is equivalent to a no clustering effect. At this time, the patient coverage is less than 80%, and the number of clusters is relatively large; if parameter *t* > 1.7, the number of clusters will be reduced to 1, and the value of providing information will be lost. According to the comprehensive indicators, when *t* = 1.5, the number of clusters is relatively moderate, with coverage > 0.94 and discrimination > 0.838, which has achieved a relatively satisfactory result.

High patient coverage rates indicate that the process models we obtain possess high generalization ability. Similarly, high discrimination rates denote good readability. Through the sensitivity analysis of clustering parameters, the effectiveness and superiority of our proposed method are verified again.

## 6. Conclusions

To better explore and improve clinical nursing pathways, we proposed a novel method called the “Fuzzy Process Mining Method” to mine clinical pathways related to disease treatment and nursing care for patients. We applied the proposed method to the EMR data of different syndrome types of acute pancreatitis. Our results show that the proposed fuzzy process mining method can effectively be used in the study of clinical nursing processes of different diseases, and can provide medical workers with different depths of knowledge. Our process model is simple and detailed in its expressiveness, can achieve high patient coverage and discrimination, and is easily adapted to simulate uncommon medical events. It can effectively avoid the spaghetti effect in the traditional process model, and effectively express the simultaneous selection of different medical operations. We performed a comparative analysis of patients with different types of acute pancreatitis. The obtained results were confirmed by clinicians, thus demonstrating our proposed method’s feasibility, portability, and high interpretability. Finally, through the sensitivity analysis of clustering parameters, the superiority of the experimental results is further verified.

In future research, we will delve into the disease optimization problem using the fuzzy process mining method proposed in this paper, and provide evidence-based interpretations for clinical nursing and treatment by combining patient clinical outcomes.

## Figures and Tables

**Table 1:** Proportions of the most common surgical operations for three types of patients.

Patient Type	Surgery	A ^1^	B ^2^	C ^3^	D ^4^	E ^5^	F ^6^	G ^7^	H ^8^	I ^9^	J ^10^
Mild Patient	SRG2	0	0	0.03	0.08	0	0.01	0	0.58	0	0.58
	SRG3	1	0	0	0	0	0	0	0	0	0
Severe Patient	SRG5	0	0.01	0.99	0	0.82	0	0	0.8	0.16	0
	SRG6	0	0.32	0.01	0.02	1	0.01	0.2	0.66	0.1	0
MOF Patient	SRG0	0	0.11	0.67	0	0.92	0	0	0.77	0.13	0

^1^ ERCP, ^2^ external drainage of pancreatic cyst, ^3^ drainage of pancreatic abscess, ^4^ debridement and drainage of necrotizing pancreatitis, ^5^ removal of pancreatic necrotic tissue, ^6^ incision and lithotomy of pancreatic duct, ^7^ internal drainage of pancreatic cyst, ^8^ lysis of intestinal adhesions, ^9^ cholecystectomy, ^10^ laparoscopic cholecystectomy.

**Table 2:** Values of coverage and discrimination when t changes.

*t*	Patient Type	Coverage	Discrimination	Number of Clusters
1	MOF Patient	0.805	0.999	10
	Severe Patient	0.826	0.999	20
	Mild Patient	0.937	1	12
1.2	MOF Patient	0.934	0.838	9
	Severe Patient	0.891	0.999	16
	Mild Patient	0.963	1	10
1.4	MOF Patient	0.887	0.982	7
	Severe Patient	0.951	0.996	14
	Mild Patient	0.976	0.997	6
1.5	MOF Patient	0.94	0.838	5
	Severe Patient	0.964	0.986	8
	Mild Patient	0.99	0.977	4
1.6	MOF Patient	0.942	0.838	4
	Severe Patient	0.979	0.982	7
	Mild Patient	0.998	0.79	2
1.7	MOF Patient	0.994	0.787	3
	Severe Patient	0.994	0.966	5
	Mild Patient	0.997	0.79	2

## Data Availability

Data are available upon request to the corresponding author/s.
